# CVD-Grown Carbon Nanotube Branches on Black Silicon Stems for Ultrahigh Absorbance in Wide Wavelength Range

**DOI:** 10.1038/s41598-020-60580-8

**Published:** 2020-03-02

**Authors:** Thanh Luan Phan, Woo Jong Yu

**Affiliations:** Department of Electrical and Computer Engineering, Sungkyunkwan University, Suwon, 16419 Republic of Korea

**Keywords:** Materials science, Synthesis and processing

## Abstract

We report a black silicon-carbon nanotube (bSi-CNT) hybrid structure for ultrahigh absorbance at wide spectral range of wavelength (300–1200 nm). CNTs are densely grown on entire bSi stems by chemical vapor deposition (CVD) through uniformly coating Fe catalyst. The bSi-CNT not only increases the surface roughness for enhancing the light suppression, but also allows the absorption of light in a wide wavelength range over the Si band gap (>1000 nm owing to 1.1 eV) due to the small band gap of CNT (0.6 eV). At short wavelength below Si band gap (<1000 nm), the absorbance of bSi-CNT shows average of 98.1%, while bSi shows 89.4%, which is because of high surface roughness of bSi-CNT that enhancing the light trapping. At long wavelength over Si band gap, the absorbance of bSi-CNT was maintained to 96.3% because of the absorption in CNT, while absorbance of bSi abruptly reduces with increase wavelength. Especially, the absorbance of bSi-CNT was showed 93.5% at 1200 nm, which is about 30~90% higher than previously reported bSi. Simple growth of CNTs on bSi can dramatically enhances the absorbance without using any antireflection coating layer. Thus, this study can be employed for realizing high efficiency photovoltaic, photocatalytic applications.

## Introduction

The intensive research on black silicon (bSi)^1,2^ over the past decade has inspired a promising approach for increasing the efficiency and reducing the manufacturing costs of many applications, including photovoltaics^3–8^, photodetectors^9,10^, and water splitting via photoelectro-chemical catalysis^11–13^. Recently, considerable effort has been directed toward enhancing the light absorbance by using Si nanowires (NWs)^14,15^, Si nanocones (NCs)^16,17^, and porous Si^18,19^, as Si has a small band gap (E_g_ = 1.1 eV) that allows for light absorption in the solar spectrum^20^. Many different methods for fabricating bSi have been introduced, such as laser texturization^21,22^, reactive-ion etching^23–25^, and metal-assisted wet etching^26,27^. However, the performance of bSi nanostructures obtained via such methods is limited by two main factors: the absorbance efficiency and the absorbance over a wide wavelength range. To overcome the first limitation, many studies have been conducted to improve the absorbance efficiency^21–27^, which is increased the fabrication cost and complex facility requirements. The second limitation leads to a low absorbance efficiency over a wide spectral range. Recently, the surface structure has been modified to enhance the efficient antireflection by coating an oxide layer via atomic layer deposition (ALD) method^23,28^, thin film deposition^29–31^ or metal nanoparticles deposition^32^. However, ALD or deposition technique may limit the applications of the Si device because the outer thin-film layer can cover whole area of Si; while, the metal nanoparticles deposition has a high production cost. On the other hand, carbon materials such as single-wall carbon nanotube (SWCNT)^33^, multi-wall carbon nanotube (MWCNT)^34,35^ or graphene^36^ as known as excellent absorption material for wide range spectral of wavelength. Here, carbon materials are not only absorbing the light, but also enhance the light trapping in vertical array structure, which attributed to achieve high optical absorption^33–36^. However, silicon based application such as solar cell or photo-electrochemical water splitting, where the practical implementations required several important aspects such are band edge energy for light absorption and charge transport, or thermodynamic at semiconductor/liquid interface^12^. Thus, wide range of wavelength operation in silicon based practical application still remains the significant technical challenges to overcome.

Herein, we report a black silicon-carbon nanotube (bSi-CNT) hybrid structure for ultrahigh absorbance at wide spectral range of wavelength (300–1200 nm). CNTs are densely grown on entire bSi side walls by chemical vapor deposition (CVD) through uniformly coating Fe catalyst on bSi. The bSi-CNT hybrid structure not only increases the surface roughness for enhancing the light suppression and trapping, but also allows the absorption of light in a wide wavelength range over the Si band gap (>1000 nm owing to 1.1 eV) due to the small band gap of CNT (0.6 eV)^37^. Therefore, the absorbance of the bSi-CNT hybrid sample was exhibited average absorbance values of 96.3% in the wavelength range of 300–1200 nm. In particular, at short wavelength below Si band gap (<1000 nm), the absorbance of bSi-CNT shows average of 98.1%, while bSi shows 89.4%, which is because of high surface roughness of bSi-CNT that enhancing the light suppression and trapping. Meanwhile, at long wavelength over Si band gap (>1000 nm), the absorbance of bSi-CNT was maintained to 96.3% because of the absorption in CNT (0.6 eV), while absorbance of bSi abruptly reduces with increase wavelength. Importantly, the absorbance of bSi-CNT was showed 93.5% at 1200 nm of wavelength, which is about 30~90% higher than previously reported bSi. Furthermore, we demonstrated the impact of the CNTs by adjusting the density of the CNTs-grown on the side of the bSi stems, where the absorbance of bSi-CNT hybrid sample was increased along to the increment of the CNT density. We proposed a simple method to integrate of CNTs and bSi, which can dramatically enhances the absorbance without using any antireflection coating layer. The results can be employed for realizing high-efficiency photodiodes, solar cells, and photocatalytic water splitting in future application devices.

## Results and Discussion

**Figure 1:**
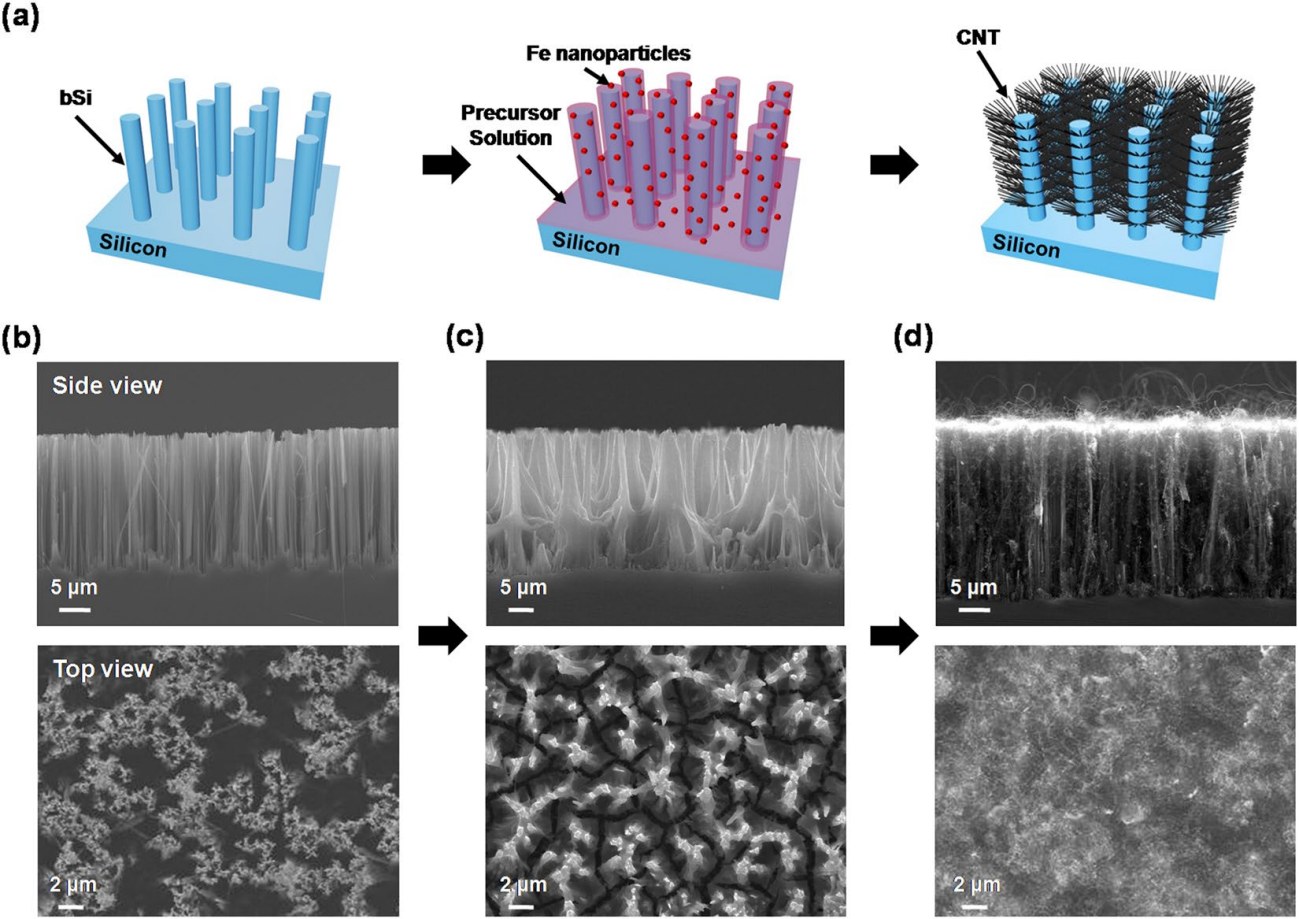
(**a**) Three-dimensional schematic illustration of the fabrication process for the bSi-CNT structure. (**b–d**) SEM images showing side views (top panels) and top views (bottom panels) of the bSi-CNT sample corresponding to (**a**).

Figure 1a shows a schematic of the fabrication process for the bSi-CNT samples. Corresponding scanning electron microscopy (SEM) images are shown in Fig. 1b–d. First, an *n*-type Si (100) substrate was immersed in a 5 M hydrofluoric acid (HF) / 0.02 M AgNO_3_ solution for 1 h at 50 °C. Then, the sample was washed with deionized (DI) water and dried with N_2_ gas^26^. The SEM image results (side and top views) are shown in Fig. 1b, where the bSi arrays are well aligned. As shown in Fig. 1c and Fig. S1, Supplementary Information, the Fe nanoparticle was uniformly deposited on the side of the bSi via electro-deposition method. The deposition was performed at room temperature in a solution containing of 5 g of FeCl_3_ and 10 g of NH_4_Cl in 100 mL of DI water for 1 min under an applied voltage (2 V) and current (0.01 A). The distance between the cathode (Pt electrode) and the anode (bSi) was 3 cm. The sample was rinsed in DI water for 5 min and then dried with N_2_ gas. Here, the top part of bSi were shrink due to the precursor solution under deposition process. Next, a CNT random network was synthesized on a bSi substrate via CVD method with methane (CH_4_) as the carbon source. The bSi substrate with a Fe catalyst precursor was then placed in a horizontal 1-inch quartz tube furnace with the catalyst end facing the gas flow. The catalyst precursor was reduced in a flowing Ar/H_2_ gas mixture (200 sccm/100 sccm) at 1000 °C for 20 min, and then 10 sccm methane with 20 sccm H_2_ gas was introduced into the furnace for the growth of the CNT random network. At the end of the growth process, Ar gas (500 sccm) was applied during cooling to room temperature^38^. As shown in Fig. 1d, the CNT random network was successfully grown on the side of the bSi sample, which was confirmed by energy-dispersive X-ray spectroscopy (EDS) measurement (Fig. S2, Supplementary Information).

**Figure 2:**
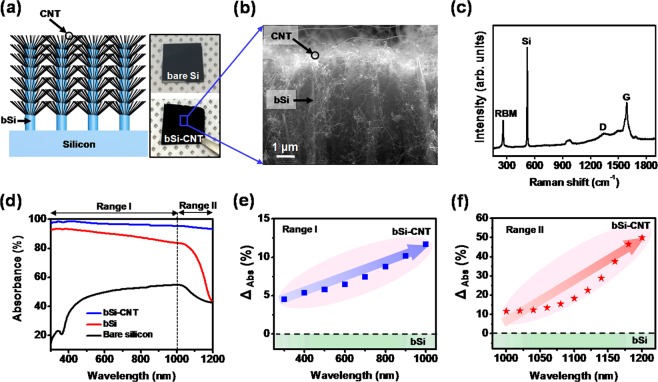
(**a**) Three-dimensional schematic illustration of the bSi-CNT hybrid structure. Insert figure are optical images of bare silicon and bSi-CNT hybrid samples. (**b**) A corresponding SEM image (cross-sectional view) of the bSi-CNT sample in (**a**). (**c**) Raman spectroscopy of bSi-CNT sample was performed at a wavelength of 532 nm. (**d**) Measured absorbance spectra of the bSi-CNT sample in the wavelength range of 300–1200 nm. The absorbance enhancement (Δ_Abs_) of the bSi-CNT compared with bSi at wavelengths of (**e**) 300–1000 nm (Range I) and (**f**) 1000–1200 nm (Range II).

Figure 2a shows a three-dimensional schematic (cross-sectional view) illustration of the bSi-CNT hybrid structure. The insert figures (right panel) are the optical image of bare silicon and bSi-CNT sample, respectively. A corresponding SEM image of the bSi-CNT sample is shown in Fig. 2b, where the CNT network was grown on bSi via CVD for 30 min. We firstly performed the Raman spectroscopy with an excitation laser having a wavelength of 532 nm, as shown in Fig. 2c. The sharp radial breathing mode (RBM) peak narrowly centered at ~260 cm^−1^, D-band at ~1340 cm^−1^, and G-band at ~1592 cm^−1^ exhibited an obvious Lorentzian line shape, which originated from the single-wall carbon nanotubes (SWCNTs)^39,40^. Using the inverse relationship (ω_RBM_ = 235.9/d_t_ + 5.5) between the RBM peak frequency (ω_RBM_) and the tube diameter (d_t_), the diameters of the SWCNTs were calculated to be approximately 1–2 nm. To further confirmation, we performed the Transmission Electron Microscopy (TEM) as in Fig. S3, Supplementary Information. As results, the brunched SWCNT of single tube with the diameter of ~2 nm at different location of samples was clearly showed. This result was consistent with the Raman spectroscopy as discussed above. Additionally, a Si peak was observed at ~520 cm^−1^, which consistent with the composition of the Si-CNT sample^41^.

The optical absorbance measurements were performed over a wide range of wavelengths (300–1200 nm), as shown in Fig. 2d. Si can usually absorb light with wavelengths of <1000 nm, corresponding to the single-crystalline Si energy band gap (E_g_ = 1.1 eV)^20^. The absorbance of bare Si samples (black line) exhibited a slight increase between the wavelengths of 300 and 400 nm, remained stable (~50%) from 400 to 1000 nm (Range I), and significantly decreased from 1000 to 1200 nm (Range II). The bSi arrays (red line) exhibited far higher optical absorbance than the bare Si over the wavelength range of 300–1200 nm. In particular, at a wavelength of 300 nm, the absorbance was enhanced from 16.1% to 92.6% by the bSi arrays. This is because the bSi array surface had high surface roughness, which enhanced the light suppression and trapping. At >1000 nm (Range II), the absorbance of the bSi was significantly reduced, that similar to the bare Si sample, which was related to the absorbance wavelength range of Si. Interestingly, the optical absorbance of the bSi-CNT hybrid structure (blue line) was significantly increased to 97.2% at a wavelength of 300 nm and remained stable (~96.3%) over the wide wavelength range of 300–1200 nm. At long wavelength over Si band gap (>1000 nm) (Range II), the absorbance of bSi-CNT was maintained to 96.3% because of the absorption in CNT (0.6 eV), while absorbance of bSi abruptly reduces with increase wavelength. Especially, the absorbance of bSi-CNT was showed 93.5% at 1200 nm of wavelength, which is about 30~90% higher than previously reported bSi, that will be discuss later. The unprecedented absorbance of our bSi-CNT hybrid structure is attributed to the CNTs grown on the side of the bSi stems, which not only provided a multi-internal reflection structure but also absorbed light with wavelengths over >1000 nm.

Next, we characterized the absorbance enhancement (Δ_Abs_) of the bSi-CNT hybrid sample compared with the bSi sample (without CNT) in different wavelength ranges from 300 nm to 1200 nm, as shown in Fig. 2e,f, respectively. Fig. 2e shows the average Δ_Abs_ (~7.4%) in the wavelength range of 300–1000 nm (Range I). In this wavelength range, the Δ_Abs_ was slightly increased from 4.9% to 11.2% corresponding to the wavelength of 300 nm to 1000 nm. This is because, the CNTs on the side of the bSi increased the surface roughness, which induced light trapping and enhanced the optical absorbance. Importantly, at long wavelength (Range II), the Δ_Abs_ was significantly increased from 11.2% (at 1000 nm) to 52.2% (1200 nm), as shown in Fig. 2f. As results, a large Δ_Abs_ was achieved at the Range II, which attributed to the effect of CNT at long wavelength (>1000 nm), where the CNTs not only functioned as absorbance materials (E_g_ = 0.6 eV) but also formed artificial branches to increase the surface roughness, which enhancing the light suppression and trapping; to yield high absorption. Thus, the absorbance enhancement provides strong evidence for the contribution of the CNTs in our bSi-CNT hybrid structure.

**Figure 3:**
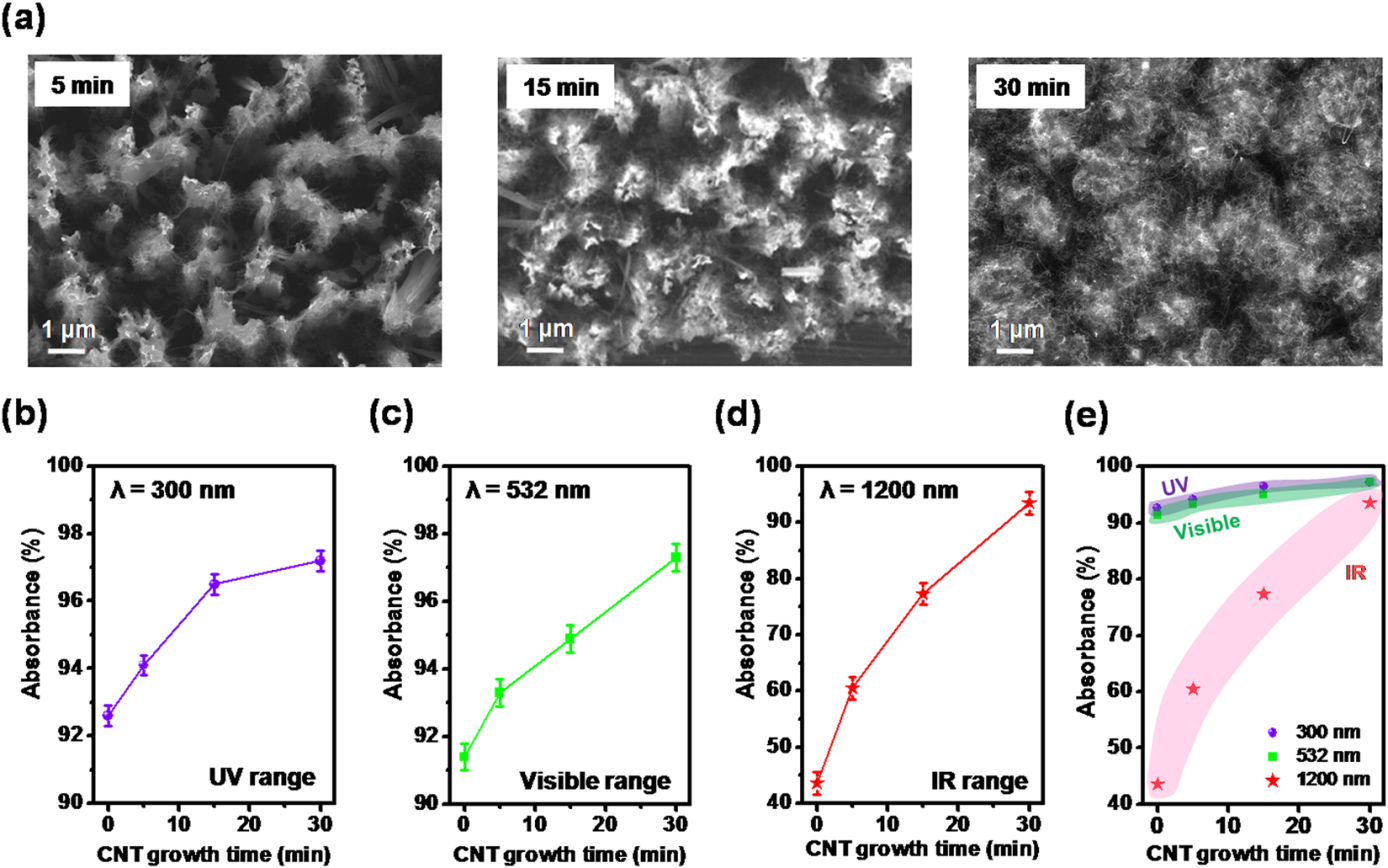
(**a**) SEM images showing the dependence of the CNT density on the CVD growth time: 5, 15, and 30 min, respectively. The absorbance versus the CNT growth time for different excitation wavelengths: (**b**) λ = 300 nm (UV range), (**c**) λ = 532 nm (Visible range), and (**d**) λ = 1200 nm (IR range). (**e**) Comparison of absorbance along to the CNT growth time at different wavelength.

To further illustrate the importance of the CNTs, we analyzed the optical absorbance with respect to the density of the CNT network in the bSi-CNT hybrid structure. Figure 3a shows SEM images (top view) of CNTs grown on bSi at various growth times from 5, 15, to 30 min, respectively. According to the SEM results, the density of the CNTs on the bSi stems was increased with the increment of CVD growth time. Together, the mass change of bSi-CNT hybrid sample due to the catalyst reduction along to the CNT growth was quantitatively calculated as shown in Fig. S4, Supplementary Information. Next, we investigated the optical absorbance of the bSi-CNT sample at 300 nm [ultraviolet (UV) range], 532 nm (visible range), and 1200 nm (IR range). As shown in Fig. 3b,c, the optical absorbance of the bSi-CNT sample was slightly increased (by 3.2% in the UV range and 4.2% in the visible range) when the CNT density increased (growth time increased from 5 to 30 min). Meanwhile, the optical absorbance in the IR range exhibited a large increase (by up to 33.6%) at 30 min, indicating the significant effect of the CNTs on the absorbance in the IR range (Fig. 3d). Figure 3e shows the comparison of absorbance along to the CNT growth time at several of wavelength (300, 532 and 1200 nm). As results, at short wavelength (300 nm and 532 nm), the absorbance of bSi-CNT obtained a slightly increased by 4.6% (300 nm-UV range) and 5.9% (532 nm-Visible) compared to the only bSi (without CNT, 0 min) at 30 min. Whereas, at long wavelength (1200 nm), the absorbance of bSi-CNT was dramatically increased up to 49.9% at 30 min growth compare to only bSi (without CNT). Thus, it is worth to note that, the CNT density strongly influenced the optical absorbance of the bSi-CNT over the wide wavelength range of 300–1200 nm, which is matched with the above discussion.

To validate the proposed device model and the high performance of the bSi-CNT design, we fabricated bSi with the nanocones shape. In contrast to bSi (with nanowires shape), the bSi nanocones (bSi NCs) had a cone shape with a lower surface roughness area, as shown in the SEM image of Fig. S5 (Supplementary Information). Prior to fabricate the bSi NC arrays, an *n*-type Si (100) wafer was first etched in buffer oxide etchant (BOE) to remove the native oxide layer and then immersed in a KOH (6 wt. %) and isopropyl alcohol (20%) solution at 70 °C for 30 min. Finally, CNTs were grown on the bSi NCs stems by using the same process that was used for the bSi samples (Fig. S6, Supplementary Information)^42^. To investigate the contribution of the CNTs to the bSi NCs, the absorbance was measured in the wavelength range of 300–1200 nm for bare Si (black line), bSi NCs (red line), and bSi NCs-CNT (blue line) hybrid samples (Fig. S7, Supplementary Information)). In particular, the optical absorbance of the bSi NCs exhibited a slight increase between the wavelengths of 300 and 400 nm, remained stable (~80%) from 400 to 1000 nm, and decreased significantly from 1000 to 1200 nm, that is similar to the results as discuss in Fig. 2d. However, the absorbance of the bSi NCs was significantly lower than that of the bSi because of the bSi NCs had lower light suppression and trapping. As results, the absorbance of bSi NCs-CNT hybrid sample was achieved 93.3% at a wavelength of 300 nm, which much higher than 62.2% of bSi NCs samples. This was a significantly increased from 55.9% (bSi NCs) to 89.6% (bSi NCs-CNT) at the wider range of wavelength (1200 nm). Importantly, the absorbance was remained stable (~91.3%) over the wide wavelength range of 300–1200 nm. This result was shown similar behavior to the bSi-CNT hybrid samples, where the large enhancement in the absorbance of the bSi-CNT and bSi NCs-CNT hybrid sample to the fact that the CNTs CVD-grown on the bSi stems not only absorbed light at wide wavelength (over 1000 nm, owing to the small band gap of CNT, 0.6 eV) but also provided high surface roughness for light suppression and trapping. This observation investigates that a CVD-grown CNT on bSi stems (even in difference shape) is very effective for enhancing the optical absorbance over a wide wavelength range without using any antireflection layer.

**Figure 4:**
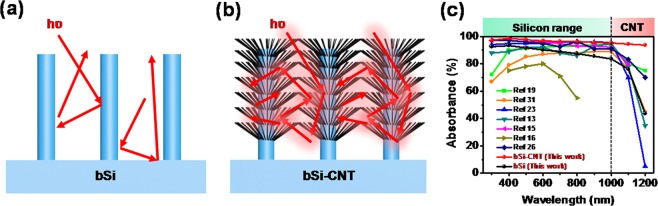
Proposed mechanism of light suppression in the (**a**) bSi and (**b**) bSi-CNT sample. (**c**) The absorbance comparisons of bSi-CNT hybrid structure with other bSi references with respect to the wavelength of 300 nm–1200 nm.

The importance of the CNTs grown on the bSi stems is schematically illustrated in Fig. 4. In general, the bSi absorbed light with a short wavelength of <1000 nm (below Si band gap), the light was confined to redirect the incident light inside bSi stems, which attribute to the absorbance enhancement (Fig. 4a). However, the absorbance of the bSi was significantly reduced at long wavelength over Si band gap (>1000 nm). This validates the CNT grown as the key factor causing the enhancement of the optical absorbance, as CNTs can absorb light at wavelengths of >1000 nm owing to the small band gap of CNT (E_g_ = 0.6 eV). Mean while, the CNTs provided an artificial branches, which increased the surface roughness at the bSi interface and increased the light confinement inside the bSi-CNT (Fig. 4b). Therefore, the average absorbance of the bSi-CNT and bSi NCs-CNT remained stable at ~96.3% (Fig. 4c) and ~91.3% (Fig. S7, Supplementary Information), respectively, at overall wavelength range of 300–1200 nm. To further comparisons between our bSi-CNT (this work) and previously reported bSi samples (references), the absorbance with respect to the wavelength from 300 nm to 1200 nm was plotted as shown in Fig. 4c. As results, the absorbance of our sample was show average of 98.1% at overall wavelength from 300 nm to 1000 nm, which is higher than all of previously references. Specially, at long wavelength over Si band gap (>1000 nm), the absorbance of bSi-CNT was showed 93.5% at 1200 nm of wavelength, which is about 30~90% higher than previously reported bSi. The unprecedented absorbance of our sample is attributed to the effect of the CVD-grown CNTs on the bSi stems, as well as the unique self-absorption of light in the IR range.

## Conclusion

In summary, we demonstrated a method for constructing a high performance bSi-CNT hybrid structure by integrating bSi stems and CVD-grown densely CNTs on bSi side walls. Here, the bSi-CNTs not only increases the surface roughness for enhancing the light suppression and trapping, but also allows the absorption of light in a wide wavelength range over the Si band gap (>1000 nm owing to 1.1 eV) due to small band gap of CNT (E_g_ = 0.6 eV). Our bSi-CNT hybrid sample achieved ~98.1% of absorbance, while bSi shows much lower 89.4% over the wavelength range <1000 nm. At long wavelength over Si band gap (>1000 nm), the absorbance of bSi-CNT hybrid sample was maintained to 96.3%, because of the absorption in CNT, while absorbance of bSi abruptly reduces with further increase wavelength up to 1200 nm. Especially, the absorbance of bSi-CNT was showed 93.5% at 1200 nm, which is about 30~90% higher than previously reported bSi. We further investigated the affect of CNT in bSi-CNT hybrid structure by adjusting the density of CNT on bSi stems, at long wavelength (1200 nm), the absorbance of bSi-CNT was dramatically increased up to ~50% at 30 min growth compare to bSi. Simple growth of CNTs on bSi can dramatically enhances the absorbance without using any antireflection coating layer. Therefore, our study can be employed for developing high performance photovoltaic and photocatalytic water-splitting applications.

## Methods

### Fabrication process for bSi

To prepare wafer scale bSi arrays, an *n*-type Si (100) substrate was immersed in a 5 M HF/0.02 M AgNO_3_ solution for 1 hat 50 °C. The bSi arrays were well aligned, and the length was adjusted by controlling the etching time. In the case of bSi (nano cones shape), an *n*-type Si (100) wafer was first etched in BOE to remove the native oxide layer and then immersed in a KOH (6 wt.%) and isopropyl alcohol (20%) solution at 70 °C for 30 min.

### Preparation of catalyst on bSi stems

Electro-deposition was used to deposit a uniform Fe catalyst on the side of the bSi on the Si substrate. The distance between the cathode (Pt electrode) and the anode (bSi) substrate was 3 cm. The deposition was performed at the room temperature in a solution containing 5 g of FeCl_3_ and 10 g of NH_4_Cl in 100 mL of DI water for 1 min under an applied voltage (2 V) and current (0.01 A). The resulting samples were rinsed in DI water for 5 min and then dried with N_2_ gas.

### Synthesis of CNTs via CVD method

The CNT random network was synthesized via catalytic CVD with methane as the carbon source. The bSi stems substrate with a catalyst precursor was placed in a horizontal 1-inch quartz tube furnace with the catalyst end facing the gas flow. The catalyst precursor was reduced in a flowing Ar/H_2_ gas mixture (200 sccm/100 sccm) at 1000 °C for 20 min, and then 10 sccm methane with 20 sccm H_2_ gas was introduced into the furnace for the growth of CNTs. At the end of the growth process, Ar gas (500 sccm) was applied during cooling to room temperature.

### Characterization

The optical absorbance characterizations of the bare silicon, bSi and bSi-CNT samples were performed using a UV-VIS/NIR Spectrophotometer (Jasco International Co., Ltd, V-670, spectral range of 300–1200 nm). Raman spectroscopy was performed using a Witec system (at a wavelength of 532 nm). SEM (JEOL, JSM-6510) images were obtained in the secondary-electron image mode at an accelerating voltage of 10 kV.

## Supplementary information

Supplementary Information.
