# Supplementary material for: CVD-Grown Carbon Nanotube Branches on Black Silicon Stems for Ultrahigh Absorbance in Wide Wavelength Range
Source: Sci Rep. 2020 Mar 2;10:3441. doi: 10.1038/s41598-020-60580-8 (PMC7049560; doi:10.1038/s41598-020-60580-8)
Supplement: Supplementary file 1 — Supplementary Information. [file 41598_2020_60580_MOESM1_ESM.docx]

**Supplementary information**

**CVD-Grown Carbon Nanotube Branches on Black Silicon Stems for Ultrahigh Absorbance in Wide Wavelength Range**

Thanh Luan Phan^+^, Woo Jong Yu^+,^***

Department of Electrical and Computer Engineering, Sungkyunkwan University, Suwon 16419, South Korea.

***Email: [micco21@skku.edu](mailto:micco21@skku.edu) (W.J.Y)

^+^These authors contributed equally to this work.

**S1. The size distribution of Fe nanoparticles catalyst.**

To verify the Fe nanoparticle size distribution, we performed our sample following the description in the Method section under 5 s as SEM image [Figure S1 (a)]. As the results, the Fe nanoparticle size was shown uniformity around ~ 20 nm. Together, in relationship to the particle size of Fe after H_2_ after CVD-grown process, the SEM measurement was conducted as shown in Figure S1 (b). As the results, the Fe nanoparticle size was shown around ~ 20 nm, which are similar to the (a).


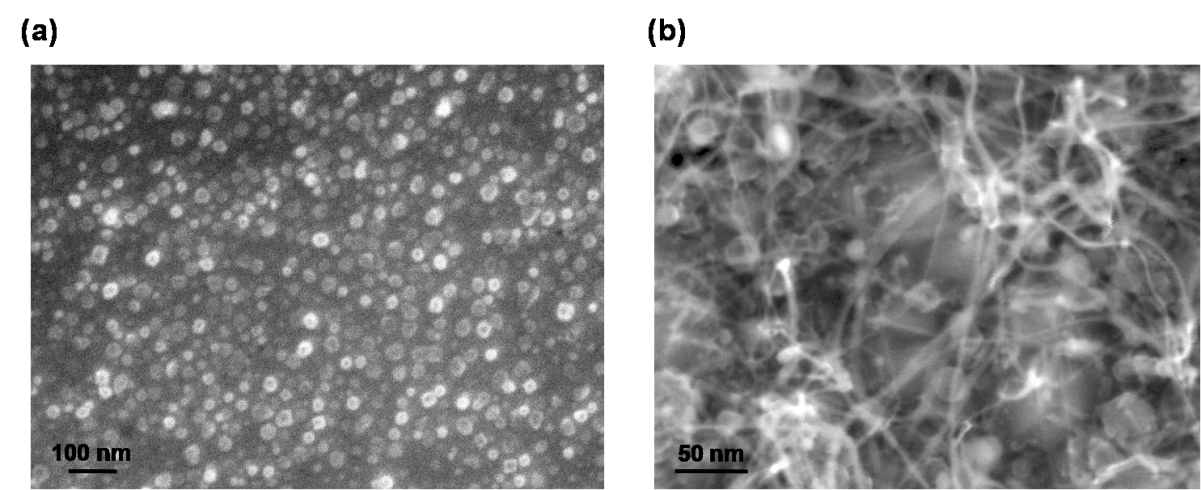


**Figure S1.** (a) The SEM image of the Fe catalyst deposition after 5 s. The size distribution of Fe nanoparticles was ~20 nm. (b) The SEM image of Fe catalyst after CVD growth process under H_2_ gas for 5 mins.

**S2. Energy-dispersive X-ray spectroscopy (EDS) spectrum of the bSi-CNT sample.**

**
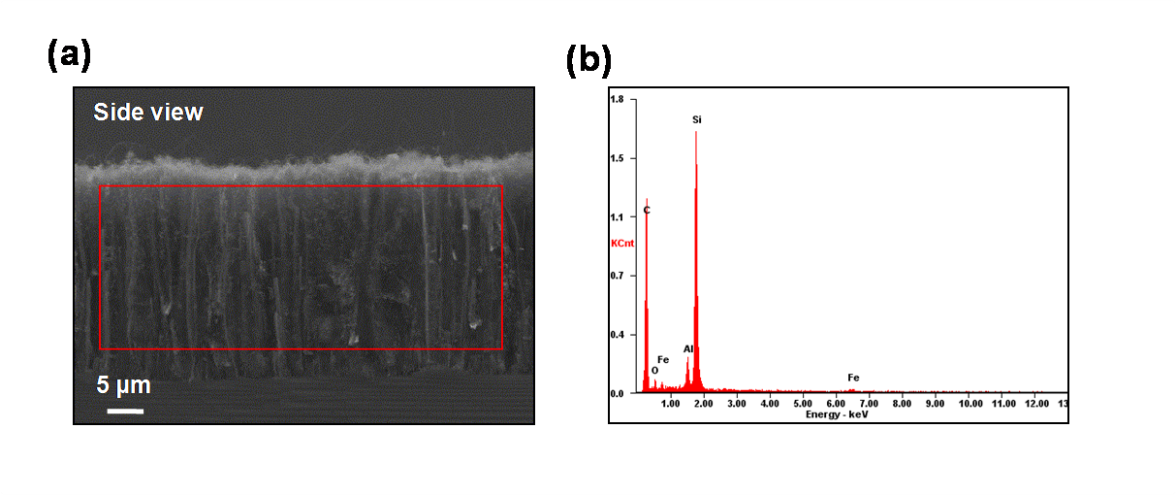
**

**Figure S2. (a)** SEM image (cross-sectional view) of the bSi-CNT sample and **(b)** EDS spectrum of the bSi-CNT sample, corresponding to the red rectangular area in (a).

**S3. The transmission electron microscopy (TEM) measurement of the CVD-grown CNT.**

**Figure S3**. The transmission electron microscopy (TEM) measurement for the CVD-grown CNT on bSi at different location of samples (a-d). The brunched SWCNT of single tube with the diameter ~ 2 nm.

**S4. The mass change of bSi-CNT along to the CNT growth.**

We are quantitatively calculating the mass change of bSi-CNT hybrid structure for before (0 min) and after CNT growth at various times of 5, 15 and 30 min via CVD method. As results, the reduction of catalyst was reducing the mass change to from 100% to 60.86%, corresponding to before (0 min) and after growth time (5 min). With increasing the CVD-grown time to 15 and 30 min, the slightly mass change was obtained to 65.83% and 69.88% for 15 and 30 min, respectively.


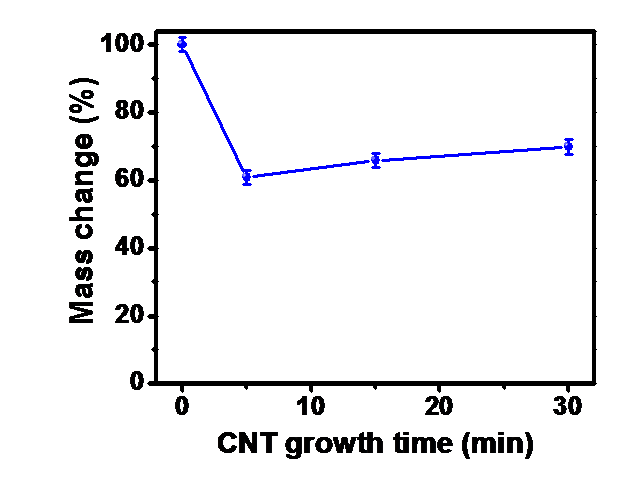


**Figure S4.** The mass change of bSi-CNT hybrid sample along to the CNT growth at various times of 5, 15 and 30 min.

**S5. Morphology of the bSi NCs structure.**

**Figure S5.** SEM image of the bSi NCs sample with **(a)** a cross-sectional view and **(b)** a top view.

**S6. Raman spectroscopy of the bSi NCs-CNT sample.**

**Figure S6. (a)** SEM image (top view) of the bSi NCs-CNT sample (CVD-grown CNTs on bSi NCs stems for 30 min). Raman spectra of the bSi NCs-CNT sample, showing the RBM peak (b) and the D and G peaks **(c)**. The inset in (c) shows the Si peak at 520 cm^-1^.

**S7. Absorbance of the bSi NCs-CNT sample in the wavelength range of 300**–**1200 nm.**

**Figure S7.** Measured absorbance spectra in the wavelength range of 300–1200 nm for bare Si, bSi NCs, and bSi NCs-CNT.
